# VEGF receptor‐2/neuropilin 1 *trans*‐complex formation between endothelial and tumor cells is an independent predictor of pancreatic cancer survival

**DOI:** 10.1002/path.5141

**Published:** 2018-09-04

**Authors:** Eric Morin, Elin Sjöberg, Vegard Tjomsland, Chiara Testini, Cecilia Lindskog, Oskar Franklin, Malin Sund, Daniel Öhlund, Sara Kiflemariam, Tobias Sjöblom, Lena Claesson‐Welsh

**Affiliations:** ^1^ Uppsala University Department of Immunology, Genetics and Pathology, Rudbeck Laboratory, Science for Life Laboratory Uppsala Sweden; ^2^ University of Oslo, Department of Hepato‐pancreato‐biliary Surgery Oslo University Hospital, Institute of Clinical Medicine Oslo Norway; ^3^ Umeå University Department of Surgery and Perioperative Sciences Umeå Sweden; ^4^ Umeå University Department of Radiation Sciences Umeå Sweden; ^5^ Umeå University Wallenberg Centre for Molecular Medicine Umeå Sweden

**Keywords:** VEGF, neuropilin 1, pancreatic adenocarcinoma, *trans*‐complex, branching

## Abstract

Unstable and dysfunctional tumor vasculature promotes cancer progression and spread. Signal transduction by the pro‐angiogenic vascular endothelial growth factor (VEGF) receptor‐2 (VEGFR2) is modulated by VEGFA‐dependent complex formation with neuropilin 1 (NRP1). NRP1 expressed on tumor cells can form VEGFR2/NRP1 *trans*‐complexes between tumor cells and endothelial cells which arrests VEGFR2 on the endothelial surface, thus interfering with productive VEGFR2 signaling. In mouse fibrosarcoma, VEGFR2/NRP1 *trans*‐complexes correlated with reduced tumor vessel branching and reduced tumor cell proliferation. Pancreatic ductal adenocarcinoma (PDAC) strongly expressed NRP1 on both tumor cells and endothelial cells, in contrast to other common cancer forms. Using proximity ligation assay, VEGFR2/NRP1 *trans*‐complexes were identified in human PDAC tumor tissue, and its presence was associated with reduced tumor vessel branching, reduced tumor cell proliferation, and improved patient survival after adjusting for other known survival predictors. We conclude that VEGFR2/NRP1 *trans*‐complex formation is an independent predictor of PDAC patient survival. © 2018 The Authors. *The Journal of Pathology* published by John Wiley & Sons Ltd on behalf of Pathological Society of Great Britain and Ireland.

## Introduction

Aberrant angiogenesis affects the progression and dedifferentiation of cancer 1. Anti‐angiogenic cancer therapy, given either as a neutralizing anti‐vascular endothelial growth factor (VEGFA) antibody (bevacizumab) or as receptor tyrosine kinase inhibitors (sunitinib and sorafenib), increases the progression‐free survival or overall survival in several cancer forms including metastatic colon cancer, advanced lung cancer, renal cancer, and hepatocellular carcinoma 2. In contrast, pancreatic ductal adenocarcinoma (PDAC) remains a dismal diagnosis, with a median overall survival of 6 months and a 5‐year survival rate of less than 5% 3, for which anti‐angiogenic therapy has shown limited benefits 4. The poor outcome is influenced by the often late‐stage diagnosis of already disseminated disease, and therapy resistance through an abundant desmoplastic stroma and dysfunctional vessels, resulting in poor perfusion and hypoxia 5. The mechanisms underlying the abnormal vasculature in PDAC have remained unknown.

Neuropilin 1 (NRP1) is a broadly expressed transmembrane glycoprotein lacking intrinsic enzymatic activity. NRP1 influences angiogenesis by forming a ternary complex with VEGFA and VEGFR2, thereby potentiating the bioactivity of VEGFR2, resulting in increased signaling and enhanced endothelial cell migration 6, 7, 8, 9. NRP1 directs internalization of VEGFR2 via clathrin‐dependent endocytosis and guides VEGFR2 intracellular trafficking through binding of the NRP1‐PDZ domain to GIPC1/synectin, an intracellular scaffold protein that in turn binds the motor protein myosin VI 10, 11. Additionally, NRP1 has been suggested to induce vascular permeability in a VEGFR2‐independent manner, through a pathway relying on the cytoplasmic domain of NRP1 12.

In a recent study, we used mouse tumor models expressing NRP1 or not, to establish the concept of a distinct, productive signaling pattern induced when VEGFR2 and NRP1 are co‐expressed on endothelial cells (denoted *cis*) compared with arrested signaling when the two molecules are expressed on adjacent endothelial (VEGFR2) and tumor (NRP1) cells (denoted *trans*). We showed that *trans*‐complex formation in these mouse tumor models results in reduced tumor angiogenesis, correlating with suppressed tumor initiation 13. The objective of the current study was to translate these findings to human cancer. Here, we studied the pattern of VEGR2 and NRP1 expression and the potential for complex formation in several different human cancers. VEGR2/NRP1 *trans*‐complex formation was identified at low density in human gastric cancer (GAC) and at high density in PDAC. The presence of VEGR2/NRP1 *trans*‐complexes in PDAC, but not in GAC tumors, was associated with decreased vessel formation, reduced cancer cell proliferation, and improved patient survival. Thus, although an overall high level of NRP1 expression in cancer is associated with an unfavorable prognosis 14, 15, 16, 17, the exact distribution of NRP1 on different cell types in the cancer dictates its effect on disease progression. Our findings highlight the clinical significance of VEGFR2/NRP1 *trans*‐complex formation in human PDAC.

## Materials and methods

Detailed materials and methods are provided in the supplementary material, Supplementary materials and methods.

### Ethics statement on mouse and human tumor samples

Animal experiments were carried out in strict compliance with the ethical permit provided by the Committee on the Ethics of Animal Experiments of the University of Uppsala, permit number C231/9.

The use of Human Protein Atlas (HPA) tumor tissue microarrays (TMAs) in this study (denoted HPA‐TMA) was covered by the HPA ethical permit (EPN Uppsala, Sweden, 2002/577, 2011/473) and an ethical permit granted to the investigators (EPN Uppsala 2007/116). All patient samples were anonymized.

Also included were pancreatic tumor tissues collected from patients undergoing Whipple resection at Oslo University Hospital, here denoted the ‘nine‐patient PDAC cohort’. Patient diagnosis was concluded after histological evaluation by a pathologist. PDAC samples were staged according to the International Union Against Cancer classification (TNM = tumor, node, metastasis). Consent documents and study protocols were approved by the Regional Committee for Medical and Health Research Ethics, Norway (REC South East, project number 2010/694a), and were in compliance with the Declaration of Helsinki, 1975.

A TMA of PDAC patient samples from 75 individuals who underwent surgery at the Umeå University Hospital between 1990 and 2009 was used for clinical analysis (here denoted the Umeå‐TMA). All individuals participating in this study provided their written informed consent. The study was conducted in accordance with the ethical standards of the Declaration of Helsinki, 1975 and was approved by the regional research ethics board of northern Sweden (Dnr 09‐175 M/2009–1378‐1331).

### T241 tumor growth

T241:EV (empty vector) or T241:NRP1 cells suspended in Matrigel were injected subcutaneously into the right and left flanks of C57Bl/6 wild‐type mice 13, 18. Tumors were dissected and processed after 7 days.

### TMAs

The HPA‐TMA used for *in situ* hybridization (ISH), immunohistochemical (IHC) staining, immunofluorescent (IF) staining, RNAscope® ISH (Advanced Cell Diagnostics, ACD, Newark, CA, USA), and proximity ligation assay (PLA) consisted of 20 different cancer types. Of these, 17 cancer types with duplicate samples from 12 patients and triplicate samples from 44 healthy tissues were analyzed in this study (i.e. thyroid cancer, head and neck cancer, and carcinoids were not included) 19, 20. The TMAs contained formalin‐fixed, paraffin‐embedded tissue cores and were sectioned to 4 μm thickness and mounted on Superfrost Plus microscope slides (Thermo Fisher Scientific, Waltham, MA, USA).

The Umeå‐TMA was constructed using cores of 1 mm diameter selected by an experienced pathologist and placed on recipient blocks using a TMA Grand Master machine (3DHISTECH Ltd, Budapest, Hungary). Three cores were included from each primary tumor and one to three cores from metastatic lymph nodes, if present. The cores were coded and randomly placed on the recipient blocks. Clinical data were retrieved from hospital charts. The observers were blinded for the clinical information during analysis of tissue staining.

### Annotation of ISH, IHC, RNAscope, and PLA

Whole‐slide scanned images, with 40× objective, of the ISH and IHC arrays were acquired using an Aperio ScanScope CS Slide Scanner system (Aperio Technologies, Vista, CA, USA). ISH signal intensity was scored using a three‐grade scale: 0 = no detectable signal; 1 = weak; and 2 = moderate. The distribution of staining in tumor cells and blood vessels was evaluated, and discrimination between structures was based on morphology. RNAscope ISH to detect *NRP1* and *KDR* (gene symbol for human VEGFR2) expression was scored on a four‐grade scale: 0 = no detectable signal; 1 = weak; 2 = moderate; and 3 = strong signal. Images were scored by one author, blind to the patient identity and clinical parameters.

VEGFR2/NRP1 PLA complexes in *trans* were defined as complexes in proximity to the endothelium (one nucleus away). Complexes that were overlapping with the endothelial staining were not included in the scoring. Occurrence of complexes in *trans* was scored on a four‐grade scale of 0–3, where 0 indicates not present; 1, one to two PLA signals per cell; 2, three to four PLA signals per cell; and 3, more than four PLA signals per cell. PLA complexes were scored blindly by two authors independently; in cases of a difference in scoring, images were re‐examined to reach consensus.

## Results

### Neuropilin 1 expression in *trans* reduces vessel number and branching *in vivo*

**Figure 1:**
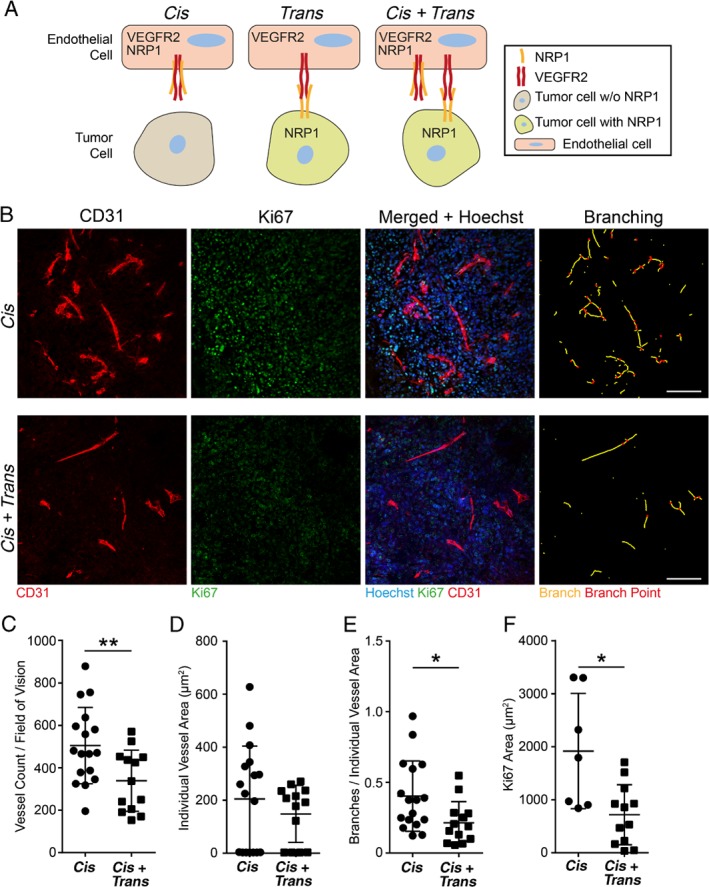
NRP1 expression in *trans* affects vascular parameters *in vivo* in murine fibrosarcoma. (A) Schematic illustration of VEGFR2/NRP1 interactions. When NRP1 is expressed on endothelial cells but not on tumor cells, VEGFR2/NRP1 complexes are formed in *cis*. When NRP1 is expressed on tumor cells but not on endothelial cells, VEGFR2/NRP1 complexes are formed in *trans*. When NRP1 is expressed on both tumor and endothelial cells, complexes are formed both in *cis* and in *trans* (*cis + trans*). (B) Representative images of CD31‐positive endothelial cells invading subcutaneous T241 fibrosarcoma lacking (T241:EV; *cis*; upper panel) or expressing NRP1 (T241:NRP1; *cis + trans*; lower panel). Panels, from left to right, show CD31‐positive endothelial cells (red); Ki67‐positive, proliferating cells (green); and merged images combined with Hoechst 33342 (blue) to visualize nuclei. The rightmost column depicts vessel branching, identifying individual vessel branches (yellow lines) and branch points (red dots). Scale bars = 100 μm. (C–F) Vessel parameters and proliferation in *cis* and *cis + trans* tumors. (C) Vessel count, (D) individual vessel area, and (E) branches per individual vessel area (see the rightmost column in B). (F) Ki67‐positive area per field of vision. Statistical analysis by Student's *t*‐test, presented as mean ±SD. *p < 0.05, **p < 0.01. One field of vision per tumor was analyzed. *Cis n* = 17 tumors; *cis + trans n* = 13 tumors.

To study the role of VEGFR2/NRP1 interactions between tumor and endothelial cells *in vivo*, we previously generated a T241 fibrosarcoma cell line stably expressing murine NRP1 (T241:NRP1) by lentivirus transduction. In parallel, T241 tumor cells, which lack endogenous expression of NRP1, were transduced with empty virus (EV) as a control 13. To facilitate even tumor establishment, T241:EV and T241:NRP1 cells were suspended in Matrigel and injected subcutaneously into the flanks of C57Bl/6 WT mice. In T241:NRP1 tumors, VEGFR2/NRP1 complexes formed both on endothelial cells (*cis*) and between tumor cells and endothelial cells (*trans*), thus creating a *cis* + *trans* condition. In T241:EV tumors, VEGFR2/NRP1 complexes were established only on endothelial cells (*cis* condition), which expressed both VEGFR2 and NRP1 endogenously (Figure 1A).

Tumors were harvested after 7 days, sectioned, and stained for Ki67, CD31, and Hoechst 33342 (Figure 1B). The *cis + trans* tumors displayed a marked reduction in vessel number compared with when NRP1 was expressed on endothelial cells alone (*cis*; Figure 1C). This is in agreement with our previous report on reduced vessel area in the presence of VEGFR2/NRP1 *trans*‐complexes 13. In contrast, vessel size, measured as individual vessel area, was not affected by the presence of NRP1 in *trans* (Figure 1D). The number of branches per vessel area was significantly reduced in the *trans* condition (Figure 1E). As tumor progression is dependent on angiogenesis, we also examined the potential consequence of VEGFR2/NRP1 *trans‐*complexes on the tumor compartment. We observed a significant reduction in tumor cell proliferation when NRP1 acted in *trans* upon endothelial cell‐expressed VEGFR2 (Figure 1F). In summary, in a mouse fibrosarcoma model, NRP1 presentation by tumor cells to the adjacent endothelium had significant effects on vessel numbers and vessel morphology, correlating with reduced tumor proliferation.

### Screening for neuropilin 1 expression in tumor cells and tumor vasculature

To investigate if the VEGFR2/NRP1 interaction can be established also in human cancer, we performed an ISH screen using the HPA‐TMA (see the Materials and methods section), analyzing duplicate tissue samples from 17 different types of cancer with 12 patients per disease 19, 20. Two independent *NRP1* riboprobes of 500–600 nucleotides 21 were used on consecutive tissue sections; a sense probe was used as a negative control (supplementary material, Figure S1A, B). The intensity of the *NRP1* signal was scored in blood vessels, in tumor cells or both, based on a graded scale from no to weak and moderate intensity (Table 1).

**Table 1:** *NRP1* ISH screening in 17 cancer types

Tumor type	Total *n*	NRP1: tumor cell expression (%)* Intensity score†			NRP1: vascular expression (%) Intensity score			NRP1: tumor and vascular expression (%)
		0	1	2	0	1	2	
Bladder	10	60	40	0	90	10	0	0
Breast	11	73	27	0	100	0	0	0
Cervix	11	82	18	0	82	9	9	0
Colorectal	10	60	40	0	80	20	0	10
Corpus	11	64	18	18	91	9	0	9
Glioma	12	75	25	0	83	17	0	8
Kidney	12	83	17	0	83	17	0	17
Liver	11	36	64	0	82	18	0	18
Lung	12	83	8	8	100	0	0	0
Lymphoma	12	83	17	0	67	33	0	0
Malignant melanoma	12	92	8	0	83	17	0	18
Ovary	11	73	27	0	91	9	0	0
Pancreas	12	42	50	8	50	50	0	33
Prostate	10	100	0	0	100	0	0	0
Squamous cell carcinoma	9	33	44	22	89	11	0	11
Stomach	8	25	63	13	38	63	0	63
Testis	8	25	63	13	75	25	0	13

* Duplicate tumor samples from up to 12 patients per cancer type scored for *NRP1* expression intensity in tumor cells and in endothelial cells presented as a percentage of the positive samples of the total.

† Intensity score ranged from 0 = negative to 2 = moderate. The rightmost column depicts the percentage of samples positive for NRP1 in both tumor cells and vasculature.

In the majority of tumor types, vascular *NRP1* expression was seen in less than 50% of the samples. Only pancreatic adenocarcinoma (PDAC) and gastric adenocarcinoma (GAC) showed the presence of *NRP1* transcripts in endothelial cells in more than 50% of the biopsies (Table 1).

Tumor cell *NRP1* expression was more consistently detected; 50–80% of the samples for hepatocellular carcinoma, pancreatic adenocarcinoma, squamous cell carcinoma, and testis and stomach cancers showed expression of *NRP1* transcripts in tumor cells (Table 1).

**Figure 2:**
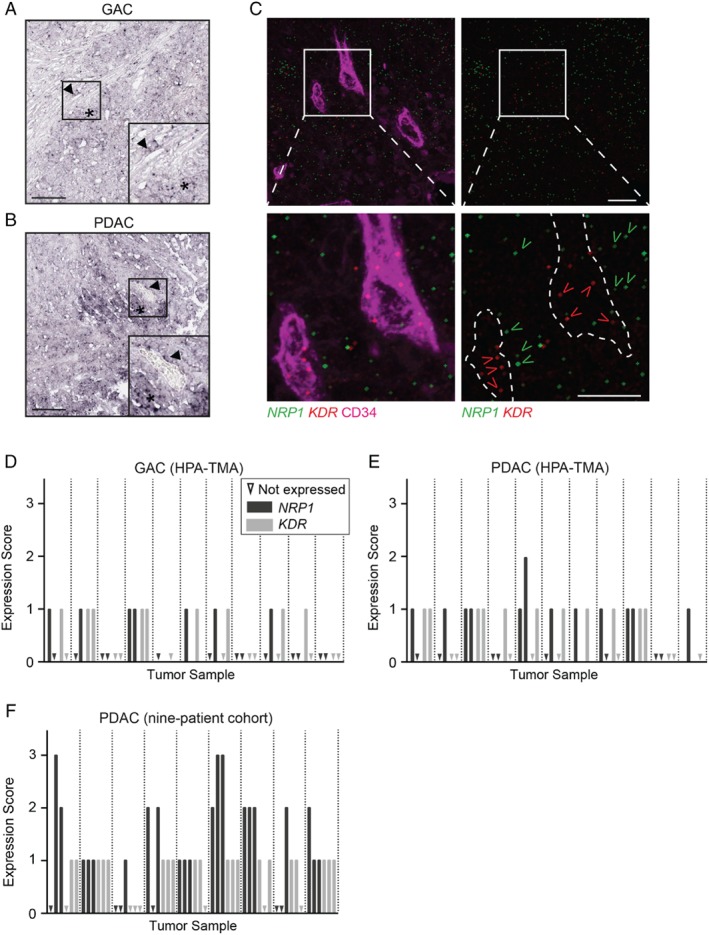
*NRP1* and *KDR* mRNA expression in human GAC and PDAC. (A, B) Representative ISH images using *NRP1* antisense probe on GAC (A) and PDAC (B) tissue sections from the HPA‐TMA. Arrowheads indicate *NRP1*‐positive blood vessels and asterisks indicate *NRP1*‐positive tumor cells. Scale bars = 200 μm. Insets show the boxed regions at higher magnification. (C) Representative images of RNAscope ISH detection of *NRP1* (green) and *KDR* (red; gene name for human VEGFR2 protein) transcripts in PDAC. Blood vessels were visualized using CD34 (magenta, left panel). Dotted line outlines CD34‐positive vessels in the right panel. Lower panels are magnifications of the boxed regions in the upper panels. Open arrowheads (green, *NRP1*; red, *KDR*) indicate examples with positive RNAscope signal. Scale bars = 10 μm. (D–F) Manual scoring of *NRP1* (dark grey) and *KDR* (light grey) expression by RNAscope ISH in GAC (D), PDAC (E) from the HPA‐TMA, and (F) the nine‐patient PDAC cohort. Scoring indicates ‘not detected’ (triangle) and increasing expression: 1 = weak; 2 = moderate; and 3 = strong. One to three fields of vision were scored for *NRP1* and *KDR* expression per tumor, separated by dotted lines.

Expression of *NRP1* in both vessels and tumor cells was detected in 11/17 cancer types, but in most cases only in a very limited number of samples, with the exception of pancreatic and gastric tumors. Moreover, only GAC and PDAC showed expression of *NRP1* in tumor cells in close proximity to blood vessels (Figure 2A, B), potentially allowing the formation of VEGFR2/NRP1 complexes in *cis* and/or *trans*.

### Multiplex mRNA expression of *NRP1* and *KDR* by RNAscope ISH in GAC and PDAC

To further investigate the potential co‐expression of NRP1 and VEGFR2 in human GAC and PDAC, we performed multiplex fluorescent ISH using the RNAscope method 22. Commercial probes against *NRP1* and *KDR* (the HUGO gene symbol for human VEGFR2), were used on the HPA‐TMA and on a separate nine‐patient PDAC cohort, along with positive (human cyclophilin B) and negative (bacterial DapB) controls (supplementary material, Figure S1C, D). Tissues were counterstained for CD34 to visualize vessels (Figure 2C). *NRP1* and *KDR* expression was evaluated by manually scoring the frequency of dots in the tumor samples, with 0 for none detected, up to 3 for strong expression (Figure 2D–F).

*NRP1* and *KDR* transcripts were detected in a majority of tumors (at least one positive biopsy core per tumor) of both GAC (6/11 and 7/11 tumors for *NRP1* and *KDR*, respectively) and PDAC (9/11 and 8/11 tumors, respectively) in the HPA‐TMA. Both *NRP1* and *KDR* were more abundantly expressed in PDAC than in GAC tumors. *NRP1* expression was present in both endothelial cells and surrounding tumor cells. Most tumors expressing *NRP1* also displayed *KDR* transcripts in endothelial cells (Figure 2D, E). In the nine‐patient PDAC cohort, both *NRP1* and *KDR* were prominently expressed in most tumors (Figure 2F).

In conclusion, *NRP1* and *KDR* transcripts were detected in GAC as well as PDAC, but more frequently and at higher levels in PDAC. In agreement, comparing the mRNA expression of *NRP1* in human tumor cell lines of gastric and pancreatic origin from the Broad Institute Cancer Cell Line Encyclopedia confirmed significantly higher *NRP1* expression in pancreatic cancer cell lines than in gastric cancer cell lines (supplementary material, Figure S1E) 23.

### NRP1 expression in GAC and PDAC

**Figure 3:**
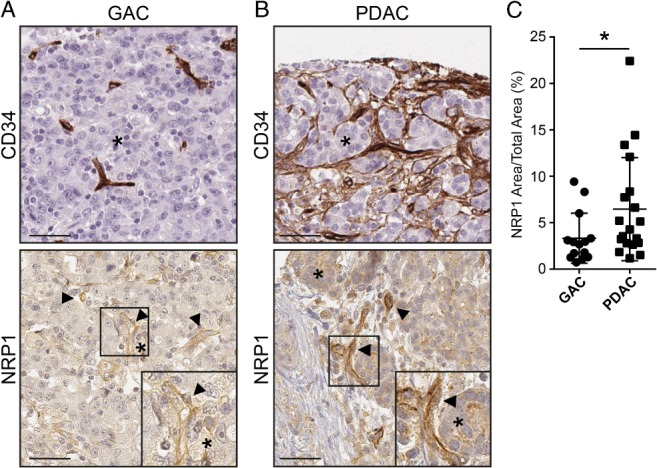
NRP1 protein expression and distribution in human GAC and PDAC. (A, B) IHC for CD34 and NRP1 on GAC (A) and PDAC (B) samples in the HPA‐TMA, counterstained with hematoxylin and eosin. Arrowheads indicate NRP1‐positive endothelial cells; asterisks indicate NRP1‐positive tumor cells adjacent to vessels. Insets highlight NRP1 expression in the vasculature and adjacent tumor cells. Scale bars = 50 μm. (C) Quantification of NRP1 expression area in GAC and PDAC tumors. Statistical analysis by Student's *t*‐test, presented as mean ±SD. *p < 0.05. GAC *n* = 15, PDAC *n* = 19 tumor samples.

To confirm and characterize NRP1 protein expression in GAC and PDAC, IHC was performed on the HPA‐TMA. NRP1 immunoreactivity was detected in endothelial cells based on morphological identification of vessels and by CD34 immunostaining run in parallel (Figure 3A, B; see magnified insets). Additionally, NRP1 was detected in the surrounding tumor tissue. The overall NRP1‐positive area was significantly higher in PDAC than in GAC, in agreement with RNA expression data (Figure 3C).

### Detection of NRP1 and VEGFR2 *trans*‐complexes in human cancer

To identify VEGFR2/NRP1 complexes in human tumors, we performed antibody‐mediated *in situ* PLA on GAC and PDAC tumor samples. *In situ* PLA allows the detection of molecular interactions on, for example, tissue sections, visualizing the localization of interactions in a complex milieu such as tumor tissues 24.

**Figure 4:**
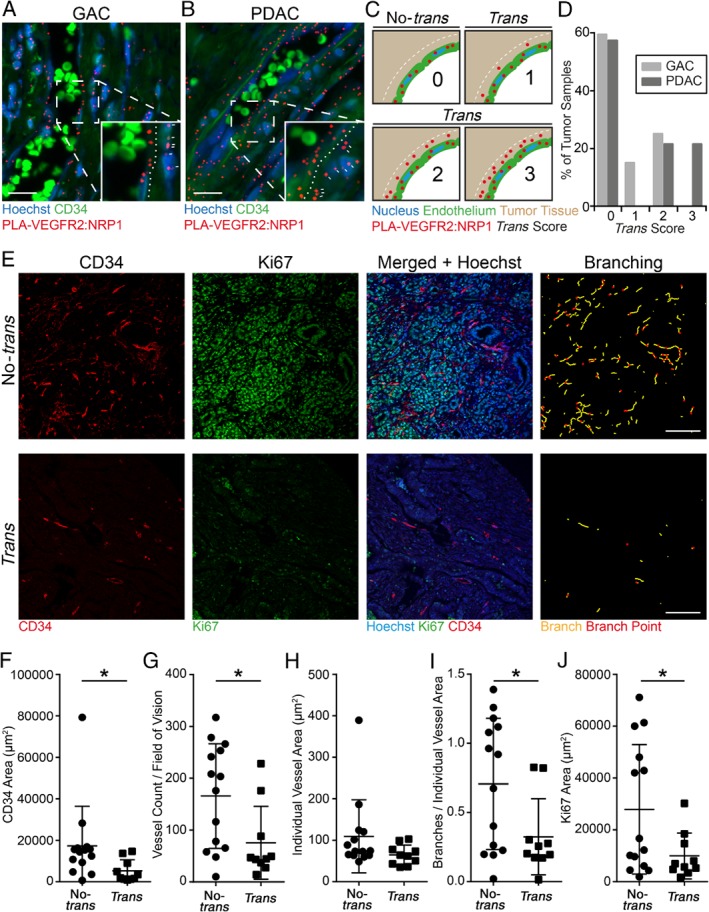
VEGFR2/NRP1 *trans*‐complexes associated with reduced tumor vasculature parameters and tumor proliferation in PDAC patients. (A, B) *In situ* PLA showing VEGFR2/NRP1 complexes (red) in GAC (A) and PDAC (B). Nuclei were counterstained with Hoechst 33342 (blue) and blood vessels were visualized using CD34 (green). Note the presence of autofluorescent erythrocytes within the vessel lumen. Insets highlight the interface of vessels and surrounding tumor tissue; dotted lines delineate the vessel–tumor border; and white dashes indicate VEGFR2/NRP1 complexes located in *trans*. Scale bars = 20 μm. (C) Schematic diagram of the scoring method for VEGFR2/NRP1 complex formation in *trans*. Tumors with complexes located adjacent to the endothelium were defined as *trans* (between the endothelium and the dotted white line). Complexes overlapping with the CD34 staining were not considered *trans*, but represented *cis‐*complexes on endothelial cells (not quantified). Scoring was as follows: 0, no PLA signal; 1, one to two PLA signals per cell; 2, three to four PLA signals per cell; and 3, more than four PLA signals per cell in the *trans* configuration. (D) Distribution of the *trans* scores in GAC and PDAC tumors from the HPA‐TMA (GAC and PDAC) and nine‐patient cohort (PDAC), presented as percentage of tumors. PDAC *n* = 24, GAC *n* = 20 tumor samples. (E) Representative images of PDAC tumors scored as no‐*trans* (upper panel) and *trans* (lower panel). Panels, from left to right, show CD34‐positive endothelial cells (red), Ki67‐positive nuclei (green), and merged images combined with Hoechst 33342 (blue). The rightmost column depicts vessel branching, identifying individual branches (yellow lines) and branch points (red dots). Scale bars = 100 μm. (F–J) Vessel parameters and tumor proliferation in PDAC tumors from the HPA‐TMA and nine‐PDAC patient cohort. (F) Total vessel area, (G) vessel number, and (H) area of individual vessels in no*‐trans* and *trans* tumors. (I) Branches per individual vessel area (see the rightmost column in E). (J) Tumor proliferation (Ki67‐positive area) per field of vision. Statistical analysis using Student's *t*‐test, presented as mean ±SD. *p < 0.05. *Trans n* = 10, no*‐trans n* = 14 tumor samples.

PLA was performed on GAC and PDAC samples in the HPA‐TMA and the nine‐patient PDAC cohort. In GAC and PDAC, complex formation was detected within the vasculature as well as between endothelial cells and adjacent tumor cells (Figure 4A, B). The specificity and reproducibility of antibodies against VEGFR2 and NRP1 were carefully controlled (supplementary material, Figure S2A–H; see supplementary material, Supplementary materials and methods for details). The presence of VEGFR2/NRP1 *trans*‐complexes was scored from 0, not present, up to a maximum of 3. *Trans*‐complexes were identified as PLA signals located outside the CD34‐positive vessel area but not more than one nucleus away from the endothelial cell (see Figure 4C for a schematic outline). Of the PDAC tumors, 43% of the samples from the HPA‐TMA and the nine‐patient cohort displayed VEGFR2/NRP1 *trans*‐complexes, and of these, 21% received the highest score of 3 (Figure 4D). Forty percent of GAC tumors displayed *trans‐*complexes, although the general frequency of complexes was markedly lower than in PDAC, and none of the tumors received a score of 3 (Figure 4D).

To correlate the presence of VEGFR2/NRP1 *trans*‐complexes to vessel parameters and tumor proliferation, we immunostained tumor samples for CD34 and Ki67 (Figure 4E). In congruence with the findings on mouse T241 tumors expressing NRP1 or not on tumor cells (Figure 1), the presence of VEGFR2/NRP1 *trans*‐complexes in PDAC correlated with reduced vascular area, vessel count, vessel branching, and tumor proliferation (Figure 4F–J), while the individual vessel area was not affected (Figure 4H). Importantly, GAC biopsies showed the same vessel parameters and tumor proliferation irrespective of the presence of VEGFR2/NRP1 *trans‐*complexes (supplementary material, Figure S3A–E), which occurred at low density.

### VEGFR2/NRP1 *trans*‐complexes correlate with improved patient survival in PDAC

**Figure 5:**
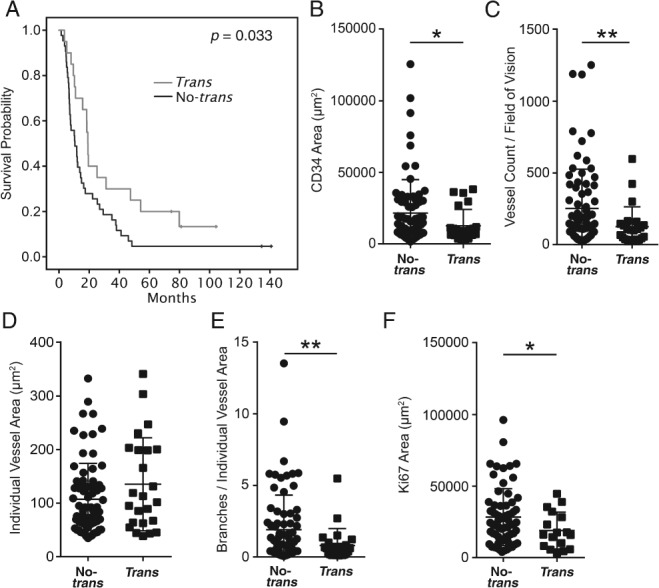
PDAC patients exhibiting NRP1 in *trans* show prolonged survival. (A) Kaplan–Meier curve of the relationship between the presence of VEGFR2/NRP1 complexes in *trans* and the overall survival of PDAC patients. The grey line represents patients positive for *trans‐*complexes (*n* = 17). The black line represents patients negative for *trans*‐complexes (*n* = 47). (B–F) Vessel parameters and tumor proliferation in PDAC tumor samples on the Umeå‐TMA. (B) Total vessel area, (C) vessel number, and (D) area of individual vessels in no*‐trans* and *trans* tumors. (E) Branches per individual vessel area. (F) Tumor proliferation (Ki67‐positive area) per field of vision. Statistical analysis using Students' *t*‐test, presented as mean ±SD. *p < 0.05, **p < 0.01. (B–E) *Trans n* = 24, no*‐trans n* = 75 tumor samples (one to three tumor samples per patient). (F) *Trans n* = 17, no‐*trans n* = 66 tumor samples (one to three tumor samples per patient).

To investigate if the presence of VEGFR2/NRP1 *trans*‐complexes is of clinical relevance, PLA for VEGFR2 and NRP1 was performed on a separate cohort (the Umeå‐TMA) consisting of 75 PDAC patients with known survival outcome. The PLA result was scored as *trans* or no‐*trans* (see Figure 4C). Kaplan–Meier analysis revealed that patients with VEGFR2/NRP1 *trans*‐complexes had a significantly improved overall survival compared with patients with no *trans*‐complexes (*p* = 0.033) (Figure 5A).

To test whether the presence of VEGFR2/NRP1 *trans*‐complexes acts as an independent marker for PDAC survival, multivariable analysis was performed including *trans* score, sex, age, tumor differentiation, and TNM stage. This analysis demonstrated that the presence of VEGFR2/NRP1 *trans*‐complexes in tumor tissue is an independent marker for overall survival in PDAC patients (HR = 0.3, *p* = 0.008) (Table 2).

**Table 2:** Multivariable analysis for overall survival in the PDAC patient cohort

Number (*n* = 75)	HR (95% CI)	*P* value
*Trans* score		
No *trans*	1	
*Trans*	0.3 (0.2–0.8)	0.008*
Sex		
Male	1	
Female	0.6 (0.3–1.2)	0.1
Age (years)		
< 60	1	
≥ 60	0.8 (0.3–2.5)	0.7
Tumor differentiation		
Well	1	
Moderate	2.0 (0.9–4.1)	0.06
Poor/undifferentiated	9.3 (2.7–31.5)	< 0.001*
TNM stage		
Ia	1	
Ib	3.8 (0.3–43.2)	0.3
IIa	2.2 (0.2–25.1)	0.5
IIb	5.5 (0.6–53.0)	0.1
III/IV	2.8 (0.2–40.2)	0.5

CI = confidence interval; HR = hazard ratio. A Cox proportional hazards model was applied using *trans* score as a categorical variable in multivariable analysis.

* *p <* 0.05 is considered significant.

Moreover, CD34‐positive vessel density (Figure 5B) and vessel count (Figure 5C) were decreased in the samples containing *trans*‐complexes, while the individual vessel area was unaffected (Figure 5D). The number of vessel branches per vessel was reduced in *trans*‐complex tumors (Figure 5E). Importantly, tumor cell proliferation was significantly reduced in the presence of VEGFR2/NRP1 *trans*‐complexes (Figure 5F). Taken together, the association of *trans‐*complexes with reduced vessel parameters and tumor proliferation was reproduced in this third cohort of PDAC patients. In addition, the increased survival of PDAC patients whose tumors contain *trans*‐complexes shows the clinical relevance of the interaction of VEGFR2 and NRP1 in cancer.

## Discussion

**Figure 6:**
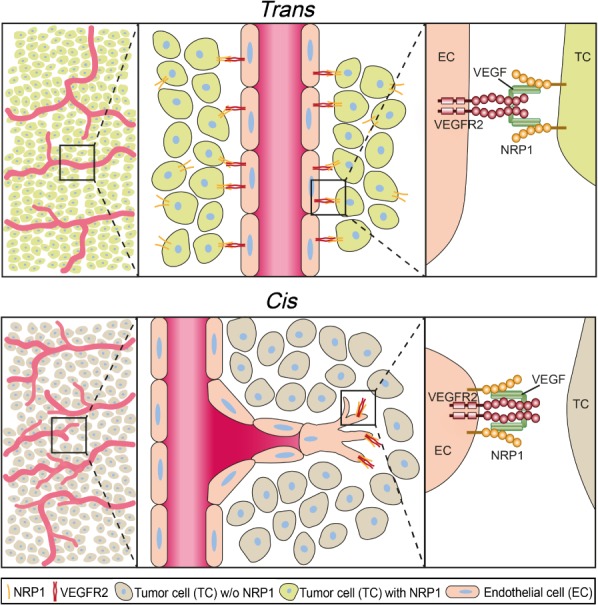
Effects of NRP1 in *cis* and *trans*. Schematic illustration of VEGFR2/NRP1 complex formation in *trans* and *cis* affecting blood vessel formation and morphology. The presence of VEGF can induce VEGFR2/NRP1 complex formation when both molecules are present on the same cell (*cis*) and when cells adjacent to the endothelial cell present NRP1 (*trans*). VEGFR2/NRP1 interaction in *trans* reduces tumor vessel branching and number, correlating with reduced tumor proliferation and improved overall survival.

High tumor vessel density correlates directly with an increased risk for liver metastasis and decreased overall survival in PDAC patients 25, 26, 27. Here, we showed that the tumor vessel density in human PDAC was reduced when NRP1 was expressed on tumor cells adjacent to blood vessels, allowing the formation of VEGFR2/NRP1 complexes in *trans* (Figure 6). Such VEGFR2/NRP1 *trans*‐complexes arrest VEGFR2 on the endothelial cell surface, interfering with productive signaling, for example, in the extracellular regulated kinase (ERK) pathway 13, ultimately resulting in suppressed tumor angiogenesis, reduced tumor cell proliferation, and prolonged patient survival.

NRP1 also interacts with ligands and receptors apart from VEGF/VEGFR2, such as semaphorins, fibroblast growth factors, and platelet‐derived growth factors, influencing their downstream signaling in different cell types 28, 29. Furthermore, NRP1 binds integrins, either indirectly via semaphorin/plexin interactions with integrins 30 or directly 31, thereby modulating cell adhesion. We cannot exclude that tumor cell‐expressed NRP1 may influence tumor angiogenesis through other interactions in addition to that with VEGFR2. However, our previous work utilizing genetic mouse models that allowed probing for NRP1's function specifically in complex formation with VEGFR2 13 forms the basis for the current work, where we have translated findings from mouse to human cancer.

To identify the expression pattern of NRP1, transcripts (ISH, RNAscope ISH), proteins (IHC), as well as protein complexes (*in situ* PLA) were studied in the intact tumor tissue. While transcript and protein levels may not strictly correlate 32, we found similar trends in the different analyses, namely that GAC and PDAC both expressed NRP1 transcript and protein in tumor cells, albeit at higher levels in PDAC.

The use of *in situ* PLA to directly show the presence of VEGFR2/NRP1 complexes in human cancer has challenges, for example in deducing the exact localization of the PLA dots. To corroborate the identification of PLA signals as corresponding to VEGFR2/NRP1 *trans*‐complexes, we studied mouse fibrosarcoma where tumor cells expressed NRP1, allowing *trans*‐complexes to be established (Figure 1). The position of PLA signals in this condition was compared with fibrosarcomas lacking NRP1 expression in tumor cells, negating the formation of *trans*‐complexes. Strikingly, the presence of VEGFR2/NRP1 *trans*‐complexes correlated with reduced vessel counts and reduced vessel branching, along with reduced tumor proliferation, in both the mouse cancer model and human PDAC (Figures 1, 4, and 5). In contrast, the expression levels of NRP1 in GAC samples were too low to allow robust formation of VEGFR2/NRP1 complexes by PLA. In accordance, tumor vessel parameters and tumor cell proliferation in GAC were not suppressed with VEGFR2/NRP1 complex formation in *trans*. These data indicate that the level of NRP1 expression in tumor cells relative to endothelial cells dictates whether *trans*‐complexes (VEGFR2/NRP1 complexes between endothelial and tumor cells) dominate over *cis*‐complexes (VEGFR2/NRP1 complexes on endothelial cells). The eventual outcome results in suppressed or augmented angiogenesis, respectively, which may influence the patient's prognosis. In agreement with the data presented here, VEGF‐dependent vessel branching has previously been shown to require VEGFR2/NRP1 *cis*‐complex formation 33, 34, 35.

NRP1 is expressed in a wide range of non‐tumor cells that may be present in a tumor, including endothelial cells, fibroblasts, and bone marrow‐derived macrophages and immune cells 36; moreover, tumor cells may also express NRP1 37. The potential prognostic effect of NRP1 expression in cancer has, however, remained unclear. In colon cancer, tumor expression of NRP1 is associated with a better prognosis 38. In cutaneous squamous cell carcinoma, NRP1 is expressed in highly differentiated tumors, suggesting that it could function as a reservoir to sequester VEGFA to the epithelial compartment, thereby limiting its bioactivity 39, reducing angiogenesis and tumor progression. On the other hand, in human glioma and breast and prostate cancer, NRP1 expression correlates with higher tumor grade and worse prognosis 14, 15, 16, and in oral squamous carcinoma, NRP1 expression has been correlated with poor prognosis and disease relapse 17. Higher levels of overall NRP1 expression correlate with poor prognosis in PDAC as well 40. Of note, the overall level of NRP1 was increased in *trans*‐complex‐containing samples (supplementary material, Figure S2H). However, based on our findings, it is essential to determine whether NRP1 is expressed in tumor cells close to endothelial cells (allowing the establishment of VEGFR2/NRP1 *trans‐*complexes) rather than the overall NRP1 expression level, as the former parameter correlates with reduced vessel density, reduced tumor proliferation, and increased patient survival.

The potential benefit of blocking NRP1 function has been tested using neutralizing anti‐NRP1 antibodies on human cancer‐xenografted mice. The anti‐NRP1 antibodies showed additive effects in slowing tumor growth when combined with anti‐VEGFA antibodies 41. As a follow‐up, early clinical trials were performed with an antibody blocking VEGFA and VEGFB binding to NRP1, in combination with VEGFA neutralization using bevacizumab. This therapy combination was, however, discontinued due to the development of proteinuria 42, 43.

Anti‐angiogenic therapy has been established preferentially in combination with chemotherapy for a number of cancers 44. Bevacizumab, inhibiting the binding of VEGFA to VEGFR2, has shown positive effects in many cancer types. However, the clinical response is very variable, which we hypothesize in part may depend on the expression pattern of NRP1. Phase III clinical trials of bevacizumab on GAC (AVAGAST) and in a separate study on PDAC patients have failed to show clinical benefit in overall survival, while, in both cases, progression‐free survival was significantly prolonged 45, 46. In the GAC AVAGAST trial, bevacizumab/chemo‐treated GAC patients with low NRP1 levels showed a significantly increased overall and progression‐free survival compared with those with higher NRP1 expression 47. A trend towards a better response to bevacizumab was also observed in breast cancer tumors with low NRP1 expression 48. An improved response to anti‐angiogenic therapy in cancer types with low NRP1 expression could be dependent on the preferential formation of VEGFR2/NRP1 *cis*‐complexes within the endothelium, promoting angiogenesis, which would be blocked by bevacizumab. In contrast, in tumors with a high degree of *trans*‐complexes, anti‐VEGF treatment would potentially interrupt the complexes and unleash VEGFR2 signaling, thereby promoting tumor angiogenesis.

To our knowledge, this is the first time that complexes between VEGFR2 and NRP1, and their correlation with vessel and tumor cell parameters, have been described in human cancer. We have shown that *trans*‐complexes correlate with reduction of several vessel parameters and tumor proliferation, as well as increased patient survival. It would be highly relevant to continue studies of this interaction in a larger cohort of PDAC patients, and also to investigate if the same effect of VEGFR2/NRP1 *trans*‐complexes can be observed in other cancer types not explored in detail here. Another important question is whether the *trans*‐group of PDAC patients shows a therapy response distinct from that of patients lacking *trans‐*complexes. We propose that the VEGFR2/NRP1 complex status could be of significant value as a prognostic marker and a potential predictive marker of anti‐angiogenic therapy in PDAC and possibly other cancer types.

## Author contributions statement

EM and LCW conceived the project. EM, ES, CT, VT, SK, TS, and LCW planned the experiments. EM, ES, CT, and SK performed experiments. EM and ES analyzed and evaluated the ISH, IH, IF, and PLA data. CLB, MS, DÖ, and OF provided critical tumor samples. EM, ES, and LCW wrote the manuscript. All authors went through and accepted the text and figures.

**SUPPLEMENTARY MATERIAL ONLINE**

**Supplementary materials and methods**

**Supplementary figure legends**

**Figure S1.** GAC and PDAC transcript expression

**Figure S2.** Antibody validation and proximity ligation assay (PLA) technical and biological controls

**Figure S3.** Vessel parameters and tumor proliferation in GAC tumors

## Supporting information


**Appendix S1.** Supplementary materials and methods

**Supplementary figure legends**

**Figure S1.** GAC and PDAC transcript expression. (A, B) Sense probe for neuropilin 1 (NRP1) shows no binding in gastric adenocarcinoma (GAC) (A) and pancreatic adenocarcinoma (PDAC) (B) tumor samples, confirming the specific binding of the corresponding antisense probes (see blue staining, Figure 2A, B). Scale bar = 200 μm. (C, D) Positive (human cyclophilin B) (C) and negative (bacterial DapB) (D) RNAscope technical controls (green). Nuclei were counterstained with Hoechst 33342 (blue). Scale bars = 10 μm. (E) Box plot of *NRP1* mRNA expression by RNAseq of cancer cell lines with gastric and pancreatic origin; data obtained from the Broad Institute Cancer Cell Line Encyclopedia (https://portals.broadinstitute.org/ccle). Statistical analysis using Student's *t*‐test, presented as mean ±SD. ***p < 0.005, stomach *n* = 35, pancreas *n* = 38.

**Figure S2.** Antibody validation and proximity ligation assay (PLA) technical and biological controls. (A) *In situ* PLA on porcine aortic endothelial (PAE) cells lacking vascular endothelial growth factor (VEGF) receptor‐2 (VEGFR2) and NRP1 expression (PAE; upper panel), or with expression of both receptors (PAE/KDR/NRP1; lower panel) treated with 75 ng/ml VEGFA. Leftmost column shows *in situ* PLA with primary antibodies against VEGFR2 and NRP1 and corresponding secondary antibodies. Middle column shows *in situ* PLA with VEGFR2 primary antibody and two appropriate secondary antibodies. Rightmost column shows *in situ* PLA with NRP1 primary antibody and two appropriate secondary antibodies. (B–E) *In situ* PLA technical controls on PDAC tissue. (B) *In situ* PLA (red dots) with VEGFR2 primary antibody and two appropriate secondary antibodies. (C) *In situ* PLA with NRP1 primary antibody and two appropriate secondary antibodies. (D, E) Negative controls with VEGFR2 (D) or NRP1 (E) primary antibody omitted, confirming the specificity of the PLA reaction. Counterstained for CD34 (green) and Hoechst 33342 (blue). Scale bars = 10 μm. (F) Representative immunofluorescence images of sectioned paraffin‐embedded PAE cells lacking (PAE, upper image) or expressing NRP1 (green) and VEGFR2 (red) (PAE/KDR/NRP1, lower image), counterstained for Hoechst 33342 (blue). Scale bar = 50 μm. (G) Representative images of PDAC tumors stained for NRP1 (green, left column) and VEGFR2 (red, middle column) merged with CD34 (cyan) and Hoechst 33342 (blue) (right column) scored as low NRP1 (upper panel) and high NRP1 (lower panel). Scale bar = 200 μm. (H) Analysis of NRP1 immunofluorescence score in PDAC patients classified as *trans* or no‐*trans*. 0 = no expression; 1 = low expression; and 2 = high expression. Statistical analysis using Mann–Whitney's test, presented as mean ±SD. ***p < 0.005. *trans n* = 8; no‐*trans n* = 10 tumor samples.

**Figure S3.** Vessel parameters and tumor proliferation in GAC tumors. (A–E) Vessel parameters and tumor proliferation in GAC tumors from the Human Protein Atlas tumor microarray (HPA‐TMA). (A) Total vessel area and (B) vessel number in no‐*trans* and *trans* groups. (C) Area of individual vessel in no‐*trans* and *trans* samples and (D) vessel branches per individual vessel area. (E) Tumor cell proliferation by Ki67 staining in GAC no‐*trans* and *trans* groups. Statistical analysis using Student's *t*‐test, presented as mean ±SD. *trans n* = 8; no‐*trans n* = 12 tumor samples.
