# Supplementary material for: VEGF receptor‐2/neuropilin 1 trans‐complex formation between endothelial and tumor cells is an independent predictor of pancreatic cancer survival
Source: J Pathol. 2018 Sep 4;246(3):311–22. doi: 10.1002/path.5141 (PMC6221118; doi:10.1002/path.5141)
Supplement: Supplementary file 1 — Appendix S1. Supplementary materials and methods [file PATH-246-311-s001.docx]

**Supplementary materials and methods**

Reference numbers refer to the main text reference list

Antibodies

Anti-neuropilin 1 (NRP1) antibody [37], clone 7130 (a kind gift from Dr Adrian Jubb, Genentech Inc, South San Franscisco, CA, USA), was used for immunohistochemistry (IHC), diluted 1:300. Anti-NRP1 (60067-1; Proteintech, Rosemont, IL, USA) was used for the proximity ligation (PLA), diluted 1:100. Anti-NRP1 (AF566; R&D Systems, Minneapolis, MN, USA) was used for immunofluorescence (IF), diluted 1:100. Anti-vascular endothelial growth factor receptor-2 (VEGFR2) antibody (AF357; R&D Systems) was used for IHC and PLA, diluted 1:100. Anti-VEGFR2 (2479; Cell Signaling Technology, Danvers, MA, USA) was used for IF, diluted 1:150. Anti-CD34 (clone QBEnd10; Agilent Technologies, Santa Clara, CA, USA) was used for IF and IHC, diluted 1:100. Dylight-650^®^ conjugated anti-CD34 (NBP2-44567C; Novus Biologicals, Littleton, CO, USA) was used for IF, diluted 1:100. Anti-CD31 (553370; BD Biosciences, Franklin Lakes, NJ, USA) was used for IF, diluted 1:100. Anti-Ki67 (ab92742; Abcam, Cambridge, UK) was used for IF at 1 µg/ml. Alexa Fluor^®^647 donkey anti-mouse, Alexa Fluor^®^555 donkey anti-rabbit, and Alexa Fluor^®^488 donkey anti-rabbit secondary antibodies (Invitrogen, Carlsbad, CA, USA) were used for IF.

*In situ* hybridization (ISH), probe generation, and hybridization

ISH probes to detect NRP1 were generated using in-house software that selects transcription regions suitable for hybridization probe design while minimizing the homology with other human transcripts. Two independent primer sets to produce 500–600 bp *NRP1* products were generated using Primer3 [49], reacting with *NRP1* nucleotides 1875–2812. To generate sense and antisense riboprobe templates, a 5′-GCGTAATACGACTCACTATAGGG-3′ - T7 promotor sequence was incorporated within the 5'-end of the forward or reverse primer, respectively. The primer sequences used to generate the two antisense *NRP1* probes were as follows: probe 1 forward 5′-GTGATAAAGTCCCCCGGATT-3′ and reverse 5′-CTACCCTGAGAATGGGTGGA-3′, probe 2 forward 5′-ACCAACCCCACAGATGTTGT-3′ and reverse 5′-CAAGGCGAAGTTTTGAGG-3′. The MegaMan (Stratagene, San Diego, CA, USA) human transcriptome library, consisting of cDNA from mRNA purified from 32 human tissues and 34 human cancer cell lines, was used for polymerase chain reactions (for details on probe generation see Kiflemariam *et al* [21]). Tissue sections were deparaffinized in xylene before hydration in a graded ethanol series (100%, 95%, and 80%, 3 min each). The hybridization was automated using Tecan GenePaint (Tecan Ag, Männedorf, Switzerland) as previously described [50,51]. The hybridization procedure was stopped by incubating slides in an EDTA-containing buffer, followed by fixation in 4% paraformaldehyde [21].

IHC

IHC staining was applied to a subset of the Human Protein Atlas tumor microarray (HPA-TMA) [[19](#_ENREF_19),[20](#_ENREF_20)], consisting of samples from cancers of the bladder, kidney, liver, pancreas, testis, and stomach. In brief, tissue sections were deparaffinized in xylene before hydration in a graded ethanol series (100%, 95%, and 80%, 3 min each). Endogenous peroxidases were blocked in 0.3% hydrogen peroxide diluted in ethanol. For antigen retrieval, a decloaking chamber (Biocare Medical, Walnut Creek, CA, USA) was used. Slides were immersed and boiled in citrate buffer, pH 6 (Lab Vision, Värmdö, Sweden), for 4 min at 125°C and then cooled to 90°C. Automated IHC was performed essentially as previously described [52], using an Autostainer XL instrument (LabVision, Gustavsberg, Sweden). Tissue sections were incubated with primary antibodies against NRP1, VEGFR2 or CD34 for 30 min and a dextran polymer visualization probe (UltraVision LP HRP polymer, LabVision) for 30 min at room temperature. Slides were developed for 10 min using diaminobenzidine (LabVision) as chromogen. All incubations were followed by rinsing in wash buffer (LabVision). Slides were counterstained with Mayer’s hematoxylin (Histolab, Gothenburg, Sweden) and mounted with coverslips using Pertex (Histolab) as mounting medium.

IF

Formalin-fixed, paraffin-embedded (FFPE) human cancer biopsies were deparaffinized using xylene, then an ethanol gradient (100%, 95%, 70%), followed by epitope retrieval in citrate buffer, pH 6, in a microwave oven for 2 × 5 min. Fresh frozen tissue sections of T241 tumors were fixed in methanol before staining. Cultured cells were fixed in 4% PFA for 10 min at room temperature before staining. Tissue sections were blocked in 10% normal donkey serum, 1% bovine serum albumin (BSA) in Tris-buffered saline (TBS), and then incubated with primary antibodies overnight at 4°C and subsequently with secondary antibodies and Hoechst 33342.

Paraffin-embedded cell pellets

Porcine aortic endothelial (PAE) cells expressing both VEGFR2 and NRP1 (stable lentiviral transfected with VEGFR2 and NRP1) or lacking the expression of both receptors [13] were detached, pelletized, and fixed with 4% PFA. Subsequently, pellets were paraffin-embedded and sectioned to 6 µm and mounted on Superfrost Plus microscope slides (Thermo Fisher Scientific, Waltham, MA, USA).

RNAscope *in situ* hybridization

Tissue slides were subjected to RNAscope^®^ multiplex fluorescent assay (Advanced Cell Diagnostics, Newark, CA, USA) for detection of RNA *in situ* according to the manufacturer’s instructions using probes against *KDR* (NM_002253.2) and *NRP1* (NM_003873.5). In brief, slides were deparaffinized in xylene and then treated with RNAscope^®^ Target Retrieval solution for 25 min at 100°C before incubation with RNAscope^®^ Protease IV. Subsequently, probes were hybridized on slides before four steps of signal amplification and counterstaining for Hoechst 33342 and CD34 (for controls see the supplementary material, Figure S1).

*In situ* proximity ligation assay (PLA)

For *in situ* PLA, tissue sections and tissue microarray were deparaffinized using xylene and an ethanol gradient (100%, 95%, 70%), followed by antigen retrieval in citrate buffer, pH 6 (Dako Target Retrieval Solution), in a pressure cooker at 125°C. Slides were subjected to *in situ* PLA according to the manufacturer’s instructions (Olink, Uppsala, Sweden). In brief, after blocking at 37°C with Duolink blocking solution to avoid unspecific antibody reactions, slides were incubated with the indicated primary antibodies in Duolink antibody diluent and washed in TBS with 0.2% Tween 20 (TBS-Tween) before applying secondary antibodies conjugated with oligonucleotides. Secondary antibodies binding to proximal epitopes are bridged by a probe to form a circular DNA structure. Rolling circle amplification was performed to amplify the ligated oligonucleotides before detection with a complementary fluorescently labeled oligonucleotide probe. Counterstaining was performed with Hoechst 33342 and an anti-CD34 antibody. As negative technical control for each experiment, to exclude unspecific activity, the same reaction was performed but with the exclusion of either primary antibody. As proof of specificity, PLA was also performed using each primary antibody separately, detected using a pair of secondary antibodies, both recognizing the primary antibody but ligated with complementary oligonucleotides. In addition to performing control PLA reactions on tumor tissue, PFA-fixed PAE cells expressing VEGFR2 and NRP1 or lacking expression of both were subjected to the same set-up of control PLA experiments (for controls see the supplementary material, Figure S2).

Image acquisition, image analysis, and statistical analysis

Microscopy was performed on an LSM 700 confocal microscope, Zeiss Axioimager 2, with Zen software (Zeiss, Oberkochen, Germany) using 20× and 63× objectives, and on a Leica confocal microscope, SP8, with Leica Application Suite X software (Leica Microsystems, Ketzlar, Germany) using 20× and 63× objectives. Processing of images was performed with ImageJ software [53] and all images were processed equally, including controls. Vessel parameters and cell proliferation analysis were automated using the open software CellProfiler™ [54]. For images of RNAscope and PLA, punctuated signals were enhanced for visualization using the CellProfiler software, with the EnhanceOrSuppressFeatures module. For statistical analysis, unless otherwise stated, an unpaired two-tailed Student’s *t*-test with Welch’s correction was performed. Probabilities of survival were estimated using the Kaplan–Meier method and log-rank test. The correlation of *trans* score with outcome was evaluated using the Cox proportional hazards regression model in uni- and multi-variable analyses. Statistical analyses were done using the SPSS software package 21.0 (IBM Corporation, Armonk, NY, USA). *P* values less than 0.05 were considered significant.
