# Supplementary figures and images for: VEGF receptor‐2/neuropilin 1 trans‐complex formation between endothelial and tumor cells is an independent predictor of pancreatic cancer survival
Source: J Pathol. 2018 Sep 4;246(3):311–22. doi: 10.1002/path.5141 (PMC6221118; doi:10.1002/path.5141)

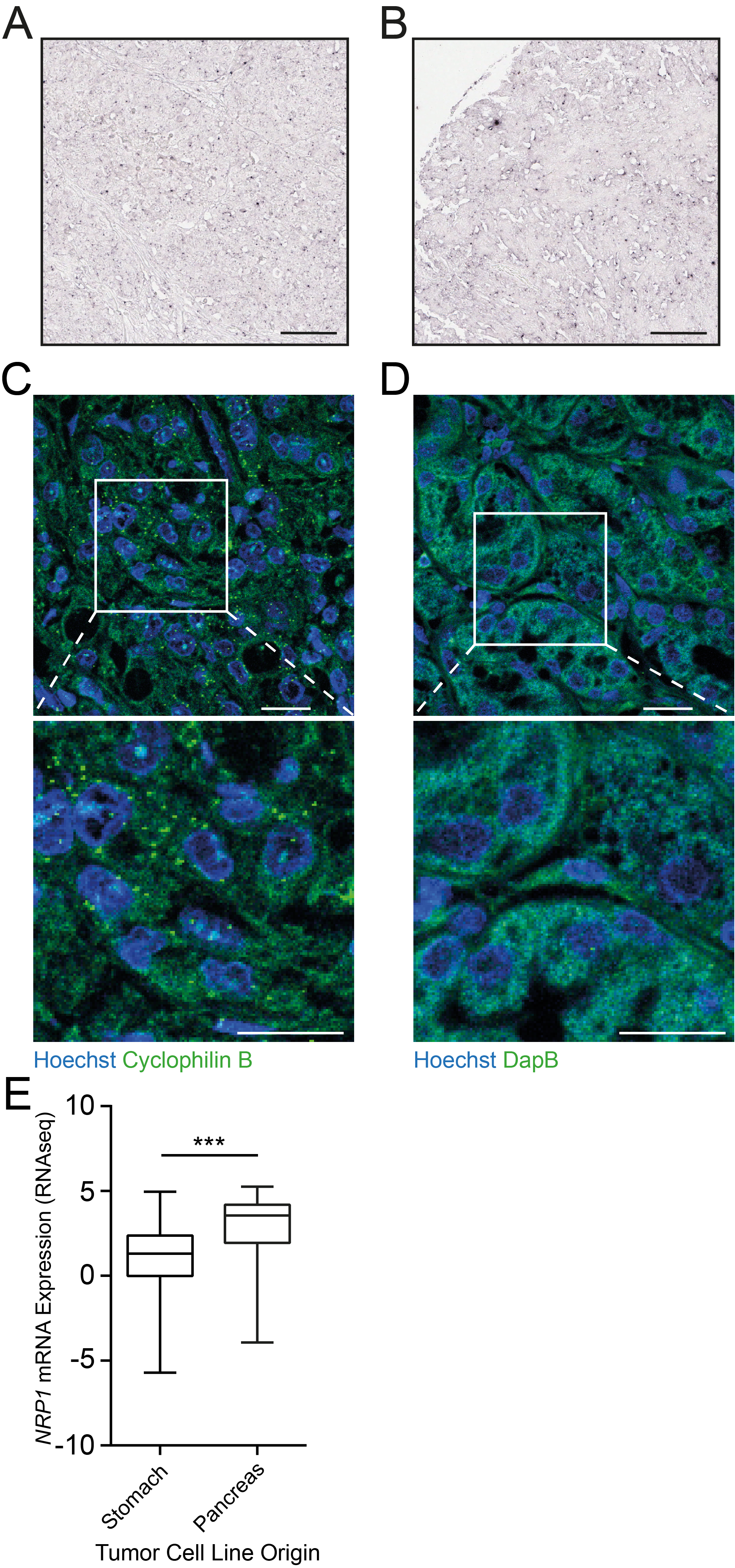

Supplement: Supplementary file 3 — Figure S1. GAC and PDAC transcript expression. (A, B) Sense probe for neuropilin 1 (NRP1) shows no binding in gastric adenocarcinoma (GAC) (A) and pancreatic adenocarcinoma (PDAC) (B) tumor samples, confirming the specific binding of the corresponding antisense probes (see blue staining, Figure 2A, B). Scale bar = 200 μm. (C, D) Positive (human cyclophilin B) (C) and negative (bacterial DapB) (D) RNAscope technical controls (green). Nuclei were counterstained with Hoechst 33342 (blue). Scale bars = 10 μm. (E) Box plot of NRP1 mRNA expression by RNAseq of cancer cell lines with gastric and pancreatic origin; data obtained from the Broad Institute Cancer Cell Line Encyclopedia (https://portals.broadinstitute.org/ccle). Statistical analysis using Student's t‐test, presented as mean ±SD. ***p < 0.005, stomach n = 35, pancreas n = 38. [file PATH-246-311-s002.tif]

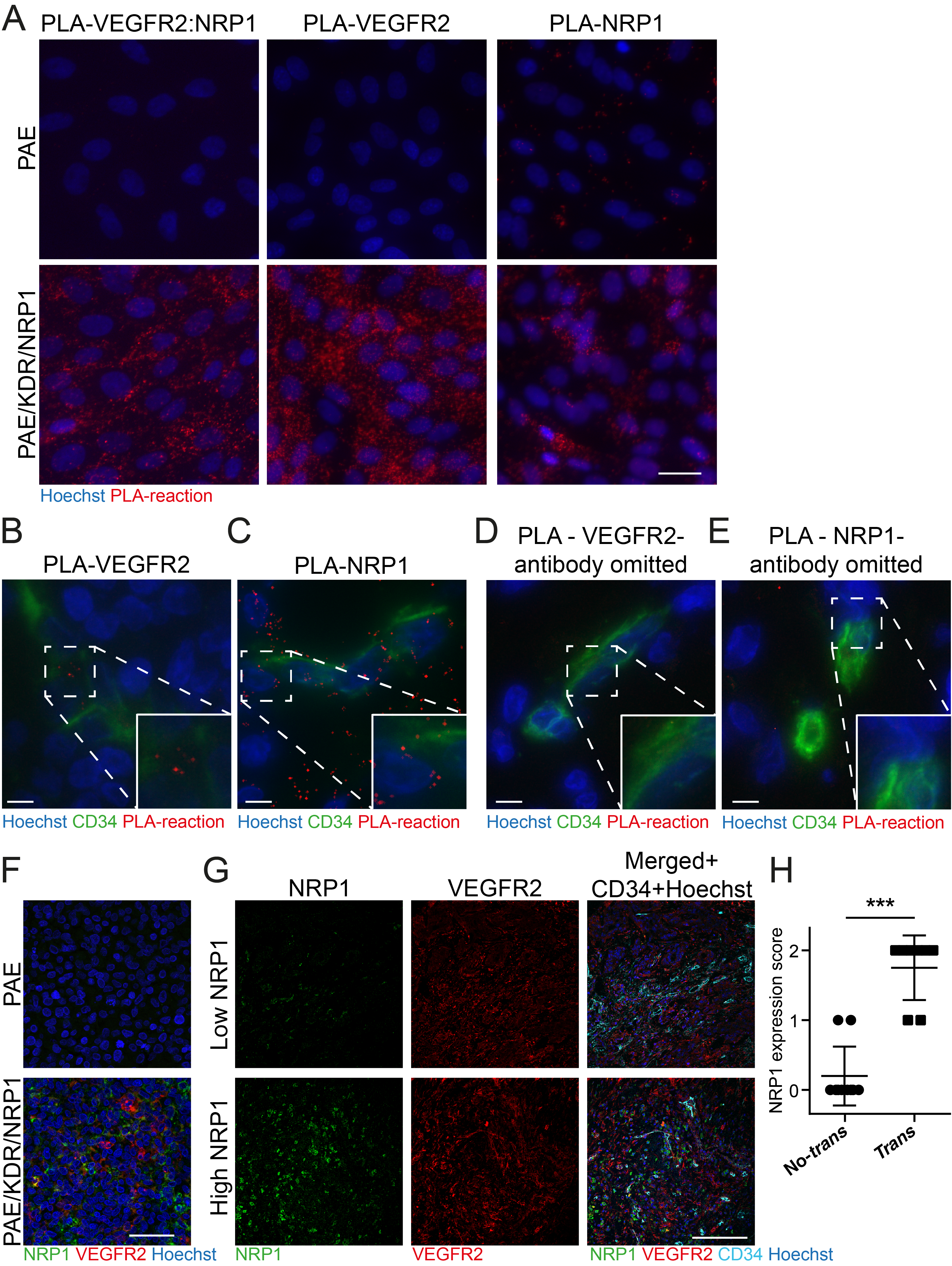

Supplement: Supplementary file 4 — Figure S2. Antibody validation and proximity ligation assay (PLA) technical and biological controls. (A) In situ PLA on porcine aortic endothelial (PAE) cells lacking vascular endothelial growth factor (VEGF) receptor‐2 (VEGFR2) and NRP1 expression (PAE; upper panel), or with expression of both receptors (PAE/KDR/NRP1; lower panel) treated with 75 ng/ml VEGFA. Leftmost column shows in situ PLA with primary antibodies against VEGFR2 and NRP1 and corresponding secondary antibodies. Middle column shows in situ PLA with VEGFR2 primary antibody and two appropriate secondary antibodies. Rightmost column shows in situ PLA with NRP1 primary antibody and two appropriate secondary antibodies. (B–E) In situ PLA technical controls on PDAC tissue. (B) In situ PLA (red dots) with VEGFR2 primary antibody and two appropriate secondary antibodies. (C) In situ PLA with NRP1 primary antibody and two appropriate secondary antibodies. (D, E) Negative controls with VEGFR2 (D) or NRP1 (E) primary antibody omitted, confirming the specificity of the PLA reaction. Counterstained for CD34 (green) and Hoechst 33342 (blue). Scale bars = 10 μm. (F) Representative immunofluorescence images of sectioned paraffin‐embedded PAE cells lacking (PAE, upper image) or expressing NRP1 (green) and VEGFR2 (red) (PAE/KDR/NRP1, lower image), counterstained for Hoechst 33342 (blue). Scale bar = 50 μm. (G) Representative images of PDAC tumors stained for NRP1 (green, left column) and VEGFR2 (red, middle column) merged with CD34 (cyan) and Hoechst 33342 (blue) (right column) scored as low NRP1 (upper panel) and high NRP1 (lower panel). Scale bar = 200 μm. (H) Analysis of NRP1 immunofluorescence score in PDAC patients classified as trans or no‐trans. 0 = no expression; 1 = low expression; and 2 = high expression. Statistical analysis using Mann–Whitney's test, presented as mean ±SD. ***p < 0.005. trans n = 8; no‐trans n = 10 tumor samples. [file PATH-246-311-s003.tif]

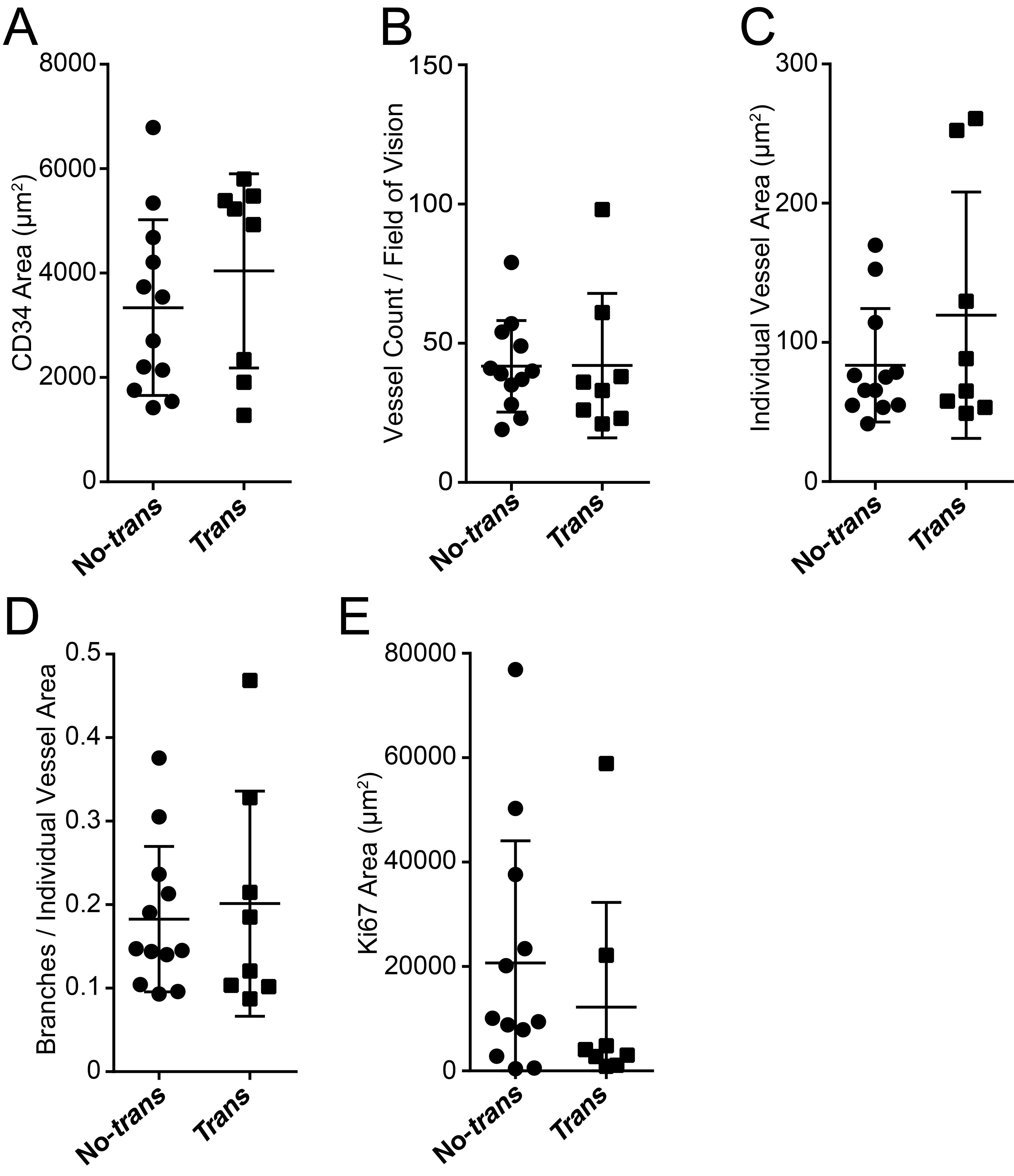

Supplement: Supplementary file 5 — Figure S3. Vessel parameters and tumor proliferation in GAC tumors. (A–E) Vessel parameters and tumor proliferation in GAC tumors from the Human Protein Atlas tumor microarray (HPA‐TMA). (A) Total vessel area and (B) vessel number in no‐trans and trans groups. (C) Area of individual vessel in no‐trans and trans samples and (D) vessel branches per individual vessel area. (E) Tumor cell proliferation by Ki67 staining in GAC no‐trans and trans groups. Statistical analysis using Student's t‐test, presented as mean ±SD. trans n = 8; no‐trans n = 12 tumor samples. [file PATH-246-311-s004.tif]
